# Clinical outcome of stereotactic body radiotherapy for primary and oligometastatic lung tumors: a single institutional study with almost uniform dose with different five treatment schedules

**DOI:** 10.1186/s13014-016-0581-2

**Published:** 2016-01-20

**Authors:** Masahiko Aoki, Yoshiomi Hatayama, Hideo Kawaguchi, Katsumi Hirose, Mariko Sato, Hiroyoshi Akimoto, Ichitaro Fujioka, Shuichi Ono, Eiki Tsushima, Yoshihiro Takai

**Affiliations:** Department of Radiology and Radiation Oncology, Graduate School of Medicine, Hirosaki University, 5 Zaifu-cho, 036-8562 Hirosaki, Aomori Japan; Department of Radiation Oncology, Southern Tohoku Proton Therapy Center, 7-172 Yatsuyamada, 963-8052 Koriyama, Fukushima Japan; Department of Physical Therapy, Graduate School of Health Sciences, Hirosaki University, 66-1 Hon-cho, 036-8564 Hirosaki, Aomori Japan

**Keywords:** Stereotactic body radiotherapy, Biologically effective dose, Lung tumor

## Abstract

**Background:**

To evaluate clinical outcomes of stereotactic body radiotherapy (SBRT) for localized primary and oligometastatic lung tumors by assessing efficacy and safety of 5 regimens of varying fraction size and number.

**Methods:**

One-hundred patients with primary lung cancer (*n* = 69) or oligometastatic lung tumors (*n* = 31), who underwent SBRT between May 2003 and August 2010, were included. The median age was 75 years (range, 45–88). Of them, 98 were judged to have medically inoperable disease, predominantly due to chronic illness or advanced age. SBRT was performed using 3 coplanar and 3 non-coplanar fixed beams with a standard linear accelerator. Fraction sizes were escalated by 1 Gy, and number of fractions given was decreased by 1 for every 20 included patients. Total target doses were between 50 and 56 Gy, administered as 5–9 fractions. The prescribed dose was defined at the isocenter, and median overall treatment duration was 10 days (range, 5–22).

**Results:**

The median follow-up was 51.1 months for survivors. The 3-year local recurrence rates for primary lung cancer and oligometastasis was 6 % and 3 %, respectively. The 3-year local recurrence rates for tumor sizes ≤3 cm and >3 cm were 3 % and 14 %, respectively (*p* = 0.124). Additionally, other factors (fraction size, total target dose, and BED_10_) were not significant predictors of local control. Radiation pneumonia (≥ grade 2) was observed in 2 patients. Radiation-induced rib fractures were observed in 22 patients. Other late adverse events of greater than grade 2 were not observed.

**Conclusion:**

Within this dataset, we did not observe a dose response in BED_10_ values between 86.4 and 102.6 Gy. SBRT with doses between 50 and 56 Gy, administered over 5–9 fractions achieved acceptable tumor control without severe complications.

## Background

The use of stereotactic body radiotherapy (SBRT) for the treatment of localized lung tumors, including early-stage non-small cell lung cancer and lung metastases, was introduced in the mid-1990s in Japan and Western countries [1–8]. Although SBRT is generally performed with higher fraction sizes, the optimal fractionation schedule for SBRT remains unclear. SBRT is associated with excellent local control and minimal toxicity; however, fatal pulmonary bleeding following radiotherapy has been reported with the use of hypofractionated regimens for centrally located tumors [9]. Although a fraction size of 12 Gy is widely used in Japan [10], we started our dose escalation study of SBRT for localized lung tumor with a fraction size of 6 Gy from May 2003 in order to avoid serious late complications. We previously published our initial clinical experience of SBRT in patients with early-stage non-small cell lung cancer and lung metastasis, using a total dose of 54 Gy administered in 9 fractions [11], and has since performed a dose escalation study with increases in fraction size of 1 Gy.

The purpose of the present study was to evaluate clinical outcomes following stereotactic body radiotherapy for localized primary and metastatic lung tumor and assess the efficacy and safety of 5 regimens with varying fraction size and number at total doses of 50–56 Gy.

## Methods

### Eligibility criteria

The initial eligibility criteria for this study were as follows: *(1)* lung cancer (T1–2N0M0) or lung metastases without active primary cancer; *(2)* maximum tumor diameter < 50 mm; *(3)* visible disease by fluoroscopy; *(4)* performance status score of ≤ 2, according to the Eastern Cooperative Oncology Group (ECOG) performance scale.

This study was approved by the institutional review board of Hirosaki University School of Medicine, and written informed consent was obtained from all patients.

### Patient and tumor characteristics

A total of 100 patients with primary or oligometastatic lung tumor underwent SBRT between May 2003 and August 2010 at our institution. All tumors were peripherally located, and all patients underwent appropriate staging studies to determine clinical diagnoses and stage. Stage IA lung cancer, stage IB lung cancer, and oligometastatic lung tumors were identified in 58, 11, and 31 patients, respectively. The primary sites of oligometastatic lung tumors in patients were as follows: lung cancer, 21; gastrointestinal cancer, 5; head and neck cancer, 3; and gynecologic cancer, 2 patients. Group A comprised patients with histopathological or cytological confirmation of disease based on the results of biopsy or cytological examination. In cases without histopathological or cytological confirmation (Group B), increases in maximum tumor diameter or standardized tumoral uptake on fluorodeoxyglucose positron emission tomography (FDG-PET CT) were required. The median patient age was 75 years (range, 45–88). Of the 100 patients, 98 (98 %) were judged to have medically inoperable disease by a multidisciplinary team of thoracic surgeons, pulmonologists, and radiation oncologists, predominantly on the basis of chronic illness or advanced age. Two patients judged to have medically operable disease refused surgery. Patient and tumor characteristics are summarized in Table 1.

**Table 1:** Patient and tumor characteristics

	Group A	Group B
Patients (*n*)	86	14
Age (y), median and range	76.0 (54–88)	70.8 (45–86)
Gender		
Male	63	7
Female	23	7
Operability		
Operable	2	0
Inoperable	84	14
Clinical diagnosis		
Primary lung cancer	66	3
Metastasis	20	11
Tumor diameter		
≤3 cm	73	13
>3 cm	13	1
Histological type		
Adenocarcinoma	58	
Squamous cell carcinoma	26	
Other	2	
Increasing tumor size		14
SUV positive		6

Abbreviations: *SUV* Standardized uptake value

Group A comprised patients with histopathological or cytological confirmation of disease based on the results of biopsy or cytological examination. Group B comprised patients without histopathological or cytological confirmation

### Treatment procedure

All patients raised both upper arms and were immobilized using a thermo-shell (ALCARE Co., Ltd., Tokyo, Japan) and a custom-made MoldCare headrest (ALCARE Co., Ltd., Tokyo, Japan) [11, 12]. Following patient immobilization, tumoral movement due to respiration of no more than 10 mm was confirmed, using an X-ray simulator (Toshiba Medical Systems Co., Ltd., Tokyo, Japan). Planning computed tomography (CT) was performed without breath-holding by a CT-simulator (Aquilion, Toshiba Medical Systems Co., Ltd., Tokyo, Japan) with 2.0 mm thickness for the identification of tumor location and calculation of treatment doses. Where tumoral movement due to respiration was 10 mm or more, planning CT was performed with breath-holding using a respiratory-monitoring apparatus (Abches, APEX Medical Inc., Tokyo, Japan). After September 2008, CT was performed using a breathing adapted technique with thickness of 2.5 mm by 4-dimensional PET/CT (Discovery ST Elite, GE Healthcare, Tokyo, Japan) and a real-time position management system (RPM gating system, Varian Medical Systems, Tokyo, Japan). A three-dimensional (3D) radiotherapy treatment-planning (RTP) machine (XiO version 4.1.1, CMS Japan, Tokyo, Japan) was used for dose calculation. Outlines of target and normal tissues (total lung, spinal cord, vertebrae) were drawn in all patients. Target margins were calculated as follows: the clinical target volume (CTV) was equal to the gross tumor volume (GTV) delineated on CT images displayed with a window level of −300 Hounsfield units (HU) and a window width of 1700 HU; the internal target volume (ITV) was calculated as CTV plus a 5–10-mm margin based on tumor movement, determined using an X-ray simulator; the planning target volume (PTV) was calculated as CTV plus with a 5-mm margin in all directions. A leaf margin of 5 mm around the PTV was also calculated.

Dose calculations were initially performed in accordance with the Clarkson’s method and the superposition method by 3D-RTP corrected for inhomogeneity. Radiotherapy was performed on 100 patients with fixed multiple coplanar and non-coplanar conformal beams by a 10-MV standard linear accelerator with EXL-20TP (Mitsubishi Electric Co., Ltd., Tokyo). Initially, 4 beams were used, and this was subsequently increased to 6 to improve dose distribution. The number of beams used was as follows: 4 in 1 patient, 5 in 7 patients, and 6 in the remaining 92 patients. The current beam arrangement consists of 3 non-coplanar oblique anterior beams in addition to 2 coplanar oblique posterior beams with 1 coplanar lateral beam. Fractionation was initially performed at a total dose of 54 Gy, administered in 9 fractions. The prescribed dose was defined as the isocenter. Fraction sizes were increased by 1 Gy, and fraction numbers were decreased by 1 fraction, for every 20 subsequent patients, thereafter. To compare the anti-tumor effects of various fractionation schedules, a biologically effective dose (BED) was utilized based on a linear-quadratic (LQ) model [13]. BED_10_ was defined as *nd* [1 + *d* / (α/β)], where *n* and *d* represent the number of fractions and fraction size, respectively, and α/β is assumed to be 10 Gy for tumor. The value of BED_10_ in our subjects were as follows: 86.4 Gy for a total dose of 54 Gy administered in 9 fractions; 95.2 Gy for 56 Gy administered in 8 fractions; 100.8 Gy for 56 Gy administered in 7 fractions; 102.6 Gy for 54 Gy administered in 6 fractions; and 100 Gy for 50 Gy administered in 5 fractions. The median overall treatment duration was 10 days (range, 5–22). Tumor location was confirmed prior to each administration with an electronic portal-imaging device (EPID). In the present study, an EPID-based setup was performed on bony anatomy.

### Follow-up and statistics

The endpoints used for evaluation were local recurrence rates and toxicity. Follow-up images were obtained at 3–6-month intervals and were used to assess tumor control. Patients were also periodically monitored by routine medical examination, during and after treatment. Local recurrence was diagnosed on the basis of enlargement of the local tumor on follow-up CT that continued for at least 6 months. FDG-PET and/or histologic confirmation was recommended when local recurrence was suspected, but this was not mandatory. Toxicities were assessed according to National Cancer Institute-Common Terminology Criteria for Adverse Events ver 4.0 (CTCAE).

All statistical analyses were performed by a competing risk analysis using R version 2.8.1 (http://www.nature.com/bmt/journal/v40/n4/full/1705727a.html). Actuarial curves were calculated by “CumIncidence.R”, according to the interval from the first date of treatment. Differences in distributions were evaluated using the log-rank test. Differences were regarded as statistically significant when *p*-value was <0.05.

## Results

The median follow-up period for all patients and survivors were 44.8 and 51.1 months, respectively. Totally, 30 of 100 patients died during the follow-up period of 3–128 months. Causes of death in these patients were other diseases in 17 patients and disease progression in 13 patients. Of the 100 patients, 70 patients were alive at the last follow-up session. The actuarial 3- and 5-year overall survival rates for all patients were 77.9 % and 68.5 %, respectively.

### Local recurrence

**Fig. 1:**
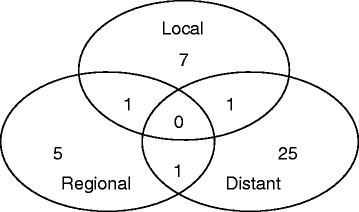
Failure patterns of the 40 disease progressions that were encountered during follow-up of the 100 patients included in this study

Of the 100 patients enrolled in this study, 9 (9 %) developed local recurrence within the follow-up period. The time to local recurrence varied between 12 and 48 months (median, 17.9). Seven of the 9 instances of local recurrence occurred within 36 months. Two of the 9 patients with local failure had additional metastases (Fig. 1). Overall, 40 patients developed progressive disease during the follow-up period and 27 patients (27 %) distant failure. Of the 27 patients with distant failure, 19 (70 %) had lung metastases outside of the radiation field.

**Fig. 2:**
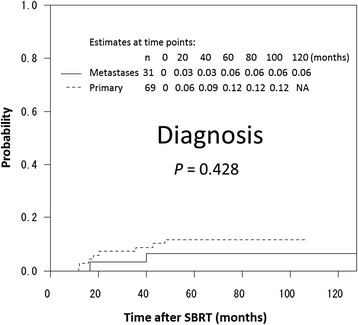
Estimated cumulative incidence curves of local recurrence rates after SBRT, according to clinical diagnosis

**Fig. 3:**
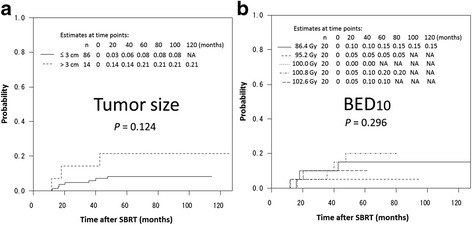
Estimated cumulative incidence curves of local recurrence rates after SBRT, according to tumor size (**a**) and BED_10_ (**b**)

The 3-year local recurrence rates for primary lung cancer and oligometastases were 6 % and 3 % (*P* = 0.428), respectively, as shown in Fig. 2. The 3-year local recurrence rates for tumor size less than 3 cm and greater than 3 cm were 3 % and 14 %, respectively (*p* = 0.124), as shown in Fig. 3a. The BED_10_ was not found to be a predictor of local recurrence, as shown in Fig. 3b. A summary of the 3-year local control rates according to each SBRT fractionation schedule is shown in Table 2. Other factors, such as fraction size and total dose, were not found to be predictors of local control.

**Table 2:** Three-year local control rates based on fractionation schedule

Fraction size	Total dose	BED_10_	Mean OTT (95 % CI)	Tumor size (n)		3-year LC
(Gy)	(Gy)	(Gy)	(days)	≤3 cm	>3 cm	(%)
6	54	86.4	14.7 (13.2–16.1)	12	8	90
7	56	95.2	11.7 (11.1–12.4)	19	1	95
8	56	100.8	10.3 (9.6–11.0)	19	1	95
9	54	102.6	8.4 (8.0–8.8)	18	2	95
10	50	100.0	7.1 (6.8–7.3)	18	2	100

Abbreviations: *BED* Biologically effective dose, *OTT* Overall treatment time, *LC* Local control

### Toxicity

Individual treatments took approximately 30 min, regardless of fractionation schedule. All patients were successfully treated without acute toxicities. The toxicities are summarized in Table 3. Grade 1 and 2 radiation pneumonitis was identified in 70 and 2 patients, respectively. In both the grade 2 pneumonitis patients, toxicity occurred 6 months after SBRT; hence, oral steroid therapy was administered, and pneumonitis resolved within 6 months.

**Table 3:** Toxicities (CTCAE criteria)

Fraction size (Gy)	6	7	8	9	10
Radiation pneumonitis (*n*)					
Grade 1	13	13	15	13	16
Grade 2	0	0	1	1	0
Radiation-induced rib fracture (*n*)					
Grade 1	3	4	7	3	5
Grade 2	0	0	0	0	0

Abbreviations: *CTCAE* Common terminology criteria for adverse events

Grade 1 radiation-induced rib fractures were identified in 22 patients. Grade 1 radiation-induced rib fractures occurred between 12 and 48 months (median, 30) after SBRT. No late adverse events greater than grade 3 were observed in the present study.

## Discussion

The results of this study indicate SBRT at total doses between 50 and 56 Gy administered in 5–9 fractions was feasible for primary lung cancer and oligometastatic lung tumors. In our study, grade 2 pulmonary toxicity was observed in 2 patients with an overall 3-year local control rate of over 90 %, equivalent to rates previously reported for SBRT [1–8]. Additionally, no significant difference was observed in BED_10_ values between 86.4 and 102.6 Gy.

Nagata Y. et al. [10] evaluated the current status of SBRT in Japan and reported fractionation schedules. According to their survey, the commonest schedules for primary lung cancer were 48 Gy administered in 4 fractions (22 institutions), followed by 50 Gy administered in 5 fractions (11 institutions), and 60 Gy administered in 8 fractions (4 institutions). The use of 48 Gy administered in 4 fractions may be the commonest schedule in Japan, as a result of the impact of a recent Japanese Phase II clinical trial (JCOG0403) [14]. However, various fractionation schedules are currently being performed in many other institutions in Japan, as there is currently a lack of consensus regarding the optimal fractionation schedules for SBRT. Therefore, BED values for tumoral and normal tissues have been utilized to compare the efficacy of various fractionation schedules, with many investigators reporting the utility of BED.

There have been several reports of the correlation between BED_10_ and local control. Onishi et al. [15] evaluated the clinical outcomes following stereotactic hypofractionated high-dose irradiation of stage I non-small cell lung carcinoma and found local control rates were better with BED_10_ ≥ 100 Gy, compared with BED_10_ < 100 Gy. Similar findings regarding the importance of BED_10_ on local control have been reported by Nagata Y. et al. [16]. BED_10_ appears to be useful in comparing the efficacy of treatment protocols with varying fraction sizes and total doses. On the other hand, Shibamoto Y, et al. [17] highlighted issues with the use of the LQ model and BED for estimating the efficacy of radiation schedules in SBRT. The LQ model has utility in the conversion of relatively low radiation doses used in conventional radiotherapy; however, it has been suggested that the LQ model is not applicable to higher daily doses or smaller fraction numbers [18]. In our study, the 3-year local control rate of over 90 % for BED_10_ < 100 Gy was equivalent to the rate of over 95 % for BED_10_ ≥ 100 Gy. This result indicates BED has no usefulness in estimating the efficacy of radiation schedules for SBRT. However, alternative mathematical models for estimating the efficacy of radiation schedules for SBRT are yet to be developed. Therefore, further research is necessary, focusing on the development of alternative mathematical models for SBRT.

Aside for the issues associated with use of the LQ model and BED, the higher local control rate in 40 patients with BED less than 100 Gy remains incompletely understood. In our study, despite 22.5 % (9/40) of tumors in the low-BED group (<100 Gy) with a maximum diameter of 3 cm or more, the 3-year local control rate was over 90 %. Additionally, in our study, the 3-year local control rate using competing risk analysis of 86 % for T2 tumors (≥3 cm) was almost equivalent to previous clinical studies that reported local control rates of 70 %–78 % for T2 tumors, following the use of higher fraction sizes (greater than 10 Gy) [19–22]. Fraction sizes were smaller, overall treatment times were longer, and fraction numbers were larger in the low-BED group. From a radiobiological standpoint, these findings suggest that during SBRT, prolonged treatment periods and larger fraction numbers may have a positive effect on local control through reoxygenation or redistribution of cancer cells during treatment. A large amount of evidence suggests tumoral reoxygenation occurs 24–72 h following irradiation [23–26]. The redistribution of cancer cells is believed to play an important role in enhancing the therapeutic effect of irradiation as a result of reoxygenation [27]. In other words, our study indicates increasing the number of fractions and extending the overall treatment duration of fractionation schedules are important for local control in addition to increasing the fraction size. However, medical expenses and the balance between local control and side effects are also important considerations for the development of optimal fractionation schedules. Jain S, et al. [28] conducted a randomized study in patients treated with four fractions of lung SBRT delivered over 4 or 11 days locking at acute toxicity and quality of life and concluded that grade 2 or higher acute toxicity was more common in the 4-day group. Although, local control rates for 4- or 11-day groups are not reported, long treatment times in Jain’s study is very interesting for future direction of SBRT schedules.

Dose/volume-effect relationships in SBRT for primary and secondary lung tumors have been discussed in recent papers. Guckenberger M, et al. [29] conducted a retrospective multi-institutional study in 399 patients with stage I non-small cell lung cancer and 397 patients with 525 lung metastases and concluded that the dose–response relationships for local tumor control in SBRT were not different between lung metastases of various primary cancer sites and between primary non-small cell lung cancers and lung metastases. Suzuki O, et al. [30] reported a dose-volume-response analysis in SBRT for early lung cancer among Japan and Western countries. The BED_10_ at PTV periphery was 102 Gy in Western countries and 83 Gy in Japan. The local control was better in Western countries for larger tumors but was similar for smaller tumors. The dose was prescribed at the isocenter in our study; dose–response relationship was not observed in tumor size and primary and oligo-metastatic lung tumors.

The current study had the following limitations. First, the study was performed without upfront power and sample size calculations. Second, analysing dose but not tumor size as risk factor for local recurrence was the primary intent of the study, and additionally, most likely by chance, tumors had been larger in the first phase of the study (at the 6 Gy level). Third, the dose was prescribed at the isocenter, and the aperture was set at 5 mm beyond PTV; the peripheral dose for PTV was different to Western approaches [30]. Fourth, all tumors included in this study were peripherally located, meaning the efficacy of these fractionation schedules for the treatment of central lesions remains unclear. Fifth, the dose calculation was changed during the study period; this possibly affected the administered doses. Finally, patient and tumor characteristic differed for each fractionation schedule because this was not a randomized study.

This study, however, provides a novel perspective on future directions for the development of optimal fractionation schedules in stereotactic body radiotherapy for patients with primary lung cancer or oligometastatic lung tumor.

## Conclusion

Regardless of fractionation schedule, stereotactic body radiotherapy with total doses between 50 and 56 Gy administered over 5–9 fractions achieved acceptable tumor control without severe complications. For stage IB primary lung cancer, however, more intensive regimen appear necessary to achieve local control. From a radiobiological standpoint, increasing the number of fractions and extending the overall treatment duration of fractionation schedules may also be important factors that influence local control.

## Abbreviations

SBRT: Stereotactic body radiotherapy
GTV: Gross tumor volume
ITV: Internal target volume
CTV: Clinical target volume
PTV: Planning target volume
SUV: Standardized uptake value
BED: Biologically effective dose
OTT: Overall treatment time
LC: Local control
CTCAE: Common terminology criteria for adverse events
